# Efficient Recovery of Noble Metal Ions (Pd^2+^, Ag^+^, Pt^2+^, and Au^3+^) from Aqueous Solutions Using *N,N'*-Bis(salicylidene)ethylenediamine (Salen) as an Extractant (Classic Solvent Extraction) and Carrier (Polymer Membranes)

**DOI:** 10.3390/membranes11110863

**Published:** 2021-11-09

**Authors:** Katarzyna Witt, Małgorzata A. Kaczorowska, Daria Bożejewicz, Włodzimierz Urbaniak

**Affiliations:** 1 Faculty of Chemical Technology and Engineering, Bydgoszcz University of Science and Technology, 3 Seminaryjna Street, PL 85326 Bydgoszcz, Poland; Malgorzata.Kaczorowska@pbs.edu.pl (M.A.K.); Daria.Bozejewicz@pbs.edu.pl (D.B.); 2 Faculty of Chemistry, Adam Mickiewicz University in Poznań, 8 Uniwersytetu Poznańskiego Street, PL 61712 Poznań, Poland; Wlodzimierz.Urbaniak@amu.edu.pl

**Keywords:** solvent extraction, polymer membranes, sorption, noble metal ions, salen, stability constant, mass spectrometry

## Abstract

This paper presents the results of the first application of *N,N'*-bis(salicylidene)ethylenediamine (salen) as an extractant in classical liquid–liquid extraction and as a carrier in membrane processes designed for the recovery of noble metal ions (Pd^2+^, Ag^+^, Pt^2+^, and Au^3+^) from aqueous solutions. In the case of the utilization of membranes, both sorption and desorption were investigated. Salen has not been used so far in the sorption processes of precious metal ions. Recovery experiments were performed on single-component solutions (containing only one type of metal ions) and polymetallic solutions (containing ions of all four metals). The stability constants of the obtained complexes were determined spectrophotometrically. In contrast, electrospray ionization high-resolution mass spectrometry (ESI-HRMS) was applied to examine the elemental composition and charge of the generated complexes of chosen noble metal ions and salen molecules. The results show the great potential of *N,N'*-bis(salicylidene)ethylenediamine as both an extractant and a carrier. In the case of single-component solutions, the extraction percentage was over 99% for all noble metal ions (molar ratio M:L of 1:1), and in the case of a polymetallic solution, it was the lowest, but over 94% for platinum ions and the highest value (over 99%) for gold ions. The percentages of sorption (%Rs) of metal ions from single-component solutions using polymer membranes containing *N,N'*-bis(salicylidene)ethylenediamine as a carrier were highest after 24 h of the process (93.23% for silver(I) ions, 74.99% for gold(III) ions, 69.11% and 66.13% for palladium(II) and platinum(II) ions, respectively), similar to the values obtained for the membrane process conducted in multi-metal solutions (92.96%, 84.26%, 80.94%, and 48.36% for Pd(II), Au(III), Ag(I), and Pt(II) ions, respectively). The percentage of desorption (%R_des_) was very high for single-component solutions (the highest, i.e., 99%, for palladium solution and the lowest, i.e., 88%, for silver solution), while for polymetallic solutions, these values were slightly lower (for Pt(II), it was the lowest at 63.25%).

## 1. Introduction

*N,N'*-bis(salicylidene)ethylenediamine (commonly known as salen, L) easily creates coordination compounds. It has been reported that this compound is a tetradentate chelating ligand, which can form mononuclear complexes by two covalent and two-coordinate covalent sites located in a planar array [1]. Due to the presence of two phenolic hydroxyl groups, which can lose the protons under appropriate conditions, the salen ligand can become a conjugate base (L^2−^). Salen and its complexes are still of interest to scientists, who study the complexation reactions between salen and various metal ions [2,3]. It is known that the complex formation process depends on multiple factors including the type of solvent used. For example, Elsherif et al. [3] investigated the stoichiometry and formation constants of complexes of salen with Cu(II) in methanol, 2-propanol, acetonitrile, tetrahydrofuran, and chloroform as nonaqueous solvents at 25 °C. They reported that the stoichiometry of the investigated complexes in all solvents were the 1:1 (M:L) type, and the order of stability was as follows: chloroform~tetrahydrofuran > 2-propanol > methanol~acetonitrile. The Cu(II) ions formed the most stable complexes with salen molecules in chloroform and the least stable in methanol. Initially, complexation properties of salen were used primarily in catalysis [4]. However, over time, the uses of salen have been extended, and one of them is separation. For example, the H_2_salen type bearing pendant tertiary amine groups [5] has been synthesized for the solvent extraction and membrane transport of transition metal salts for metal separation and purification processes. These ligands undergo a so-called zwitterionic transformation when binding metal salts, forming overall neutral assemblies, which can be transferred into non-polar, water-immiscible solvents. In addition, the recovery characteristics of copper and sulfate have been reported for a modified ligand, which shows copper-binding and thus facilitates copper-recovery and ligand-recycling. Similar solutions were also reported by Forgan et al. [6] and Galbraith et al. [7]. In turn, Dadfarnia et al. [8,9] developed a microcolumn for an online preconcentration of investigated metal ions with a flow injection-flame atomic absorption spectrometry (FI–AAS). Salen, in this case, was immobilized on surfactant-coated alumina. The method was applied to water samples, multivitamin tablets, and standard reference alloys. Kim et al. synthesized modified silica-salen(NEt_2_)_2_ [10] for the separation and concentration of the metal ions from an aqueous solution by solid-phase extraction. The synthesized salen(NEt_2_) was chemically bonded to silica gel by a diazonium coupling reaction. The procedure of adsorption was applied to three types of water samples. The authors obtained adsorption capacities and binding constants for Cu(II), Mn(II), Pb(II), and Zn(II), and the recoveries reached more than 95%. In other studies [11], the authors used a chemically modified XAD-4-salen chelating resin for the separative concentration of Cu(II), Pb(II), and Bi(III) ions from an aqueous solution. XAD-4-salen was synthesized by the reaction of salen and Amberlite XAD-4 resin. The method has been employed to determine the investigated metal ions in samples of five kinds of river water. The adsorbed metal ions were desorbed by 10 mL of 1.0 M HNO_3,_ and the total recoveries of the proposed method were more than 85% in the spiked samples in which a given amount of analytes was added. The silica derivative-salen [12] formed in a series of reactions of rice husk ash, 3-(chloropropyl)triethoxysilane, potassium iodide, and ethylenediamine also showed high potential for the extraction and removal of Ni(II), Cu(II), and Co(II) ions from aqueous solutions. Furthermore, salen has found application in so-called metal–organic frameworks (MOFs) [13,14], which are dedicated to various separation processes due to their well-defined pore size and geometry. Thus, the separation of substances can be provided on mixtures of gases, vapors, and liquid phases. In this year, salen [15] and sodium tetraphenylborate (TPB) were used as extracting agents in polymeric inclusion membranes to extract gold from aqueous solutions. The fabricated membranes showed high selectivity and reactivity toward gold. The reduction of gold on the membrane surface reached more than 90%. The presence of gold was confirmed by scanning electronic microscopy. The authors proved that the addition of salen to TPB affected the size and structure of the gold nanoparticles. Campo-Cobo et al. [16] also investigated other salen-type ligands with electron-accepting substituents on the aromatic ring as extractant agents in polymeric inclusion membranes. Salen-type ligands had higher selectivity to Au(III) than to other metals (Cu(II), Pb(II), Ca(II), Al(III), Co(II), Fe(II), Fe(III), Ni(II), Mn(II), and Zn(II)). The results showed that the percentage of extracted gold depends on the type of substituent present in the ligand’s aromatic ring, the pH of the working solution, and the membrane area. There was no effect of membrane thickness on the metal extraction. The time required to reach a certain extraction percentage decreased considerably as the membrane area increased.

In our previous paper [17], we also used salen as an extractant in liquid–liquid extraction, as a carrier during the transport across polymer inclusion membrane, and in sorption processes for the removal of Ni(II), Cu(II), and Zn(II) ions from aqueous solutions. The results of liquid–liquid extraction show that salen is a very effective extractant, especially for removing copper(II) ions from aqueous solutions. Still, its efficiency depends on its concentration in the system. Moreover, we proved that the separation of chosen metal ions using the investigated polymer membranes with salen was better during the sorption process (membrane extraction) than during the transport across those membranes.

In this paper, we present the novel investigation results of the recovery of chosen precious metal ions (Pd^2+^, Ag^+^, Pt^2+^, and Au^3+^) from aqueous solutions, using salen during the classical solvent extraction and sorption processes on polymer membranes. Establishing the proper conditions for membrane sorption is not easy as the process depends on various factors (for example, type and concentration of recovered metal ions, properties of base-polymer, plasticizer, and carrier used to fabricate the membrane, type of solvent used, pH, temperature, and process time). However, the polymer membranes have many advantages: they are effective, inexpensive, show high stability and versatility, and allow both the sorption and desorption processes to be carried out. Polymer-based non-fluid membranes are usually more stable because carrier ions in polymer membranes are chemically or physically bound within the membrane matrix. In addition, separation based on the utilization of polymer membranes is an environmentally safe alternative to solvent extraction by drastically reducing the use of toxic solvents. Therefore, the membrane extraction is defined as a sustainable green strategy [18,19,20]. The interest in the recovery of precious metals from aqueous solutions (i.e., various types of wastewater) is systematically growing due to the decline in the natural resources of these metals (ores), their intensive utilization in various industries, and the increase in the amount of waste generated (e.g., waste electronic and electrical equipment), which contain significant amounts of these metals [21]. The reuse of precious metals that originated from secondary sources is also an important part of green chemistry. In the case of known methods such as solvent extraction [22] or membrane processes dedicated to recovering precious metal ions, the key role is played by the use of a properly selected extractant/carrier that will be safe for the environment and enable sufficiently efficient recovery. The results presented in this article show the great potential of salen for the recovery of gold, silver, palladium, and platinum ions. The results of applying membrane processes are particularly promising as they are not only efficient, but are also environmentally friendly (consumption of small amounts of salen and chemical solvents), allowing for extraction and reverse extraction at low costs.

## 2. Materials and Methods

### 2.1. Reagents

All the reagents used in this work were of analytical grade purity and used without further purification. The *N,N'*-bis(salicylidene)ethylenediamine (L) was bought from Sigma-Aldrich (Poznan, Poland). The pattern metal ions (Pd^2+^, Ag^+^, Pt^2+^, and Au^3+^), nitric acid, ammonia, potassium hydroxide, chloroform, and methanol were purchased from Avantor (Gliwice, Poland). 

**Figure 1:**
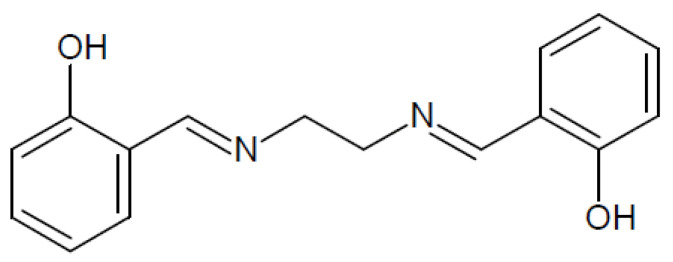
The structure of *N,N'*-bis(salicylidene)ethylenediamine (L, salen).

The structure of *N,N'*-bis (salicylidene)ethylenediamine is shown in Figure 1.

### 2.2. Mass Spectrometry

In all high-resolution mass spectrometry experiments, the mass spectrometer Q-Exactive Orbitrap (Thermo Fisher Scientific, Bremen, Germany) was used. This instrument was equipped with a TriVersa NanoMate robotic nanoflow ESI ion source (Advion BioSciences Ltd., Ithaca, NY, USA). Samples of the separated organic phases after liquid–liquid extraction (described in detail in Section 2.4) containing *N,N'*-bis(salicylidene)ethylenediamine and one type of the selected metal ion (Pd^2+^, Pt^2+^, or Au^3+^) in a molar ratio of 5:1 were diluted (1:1) in methanol. MS data were acquired in a positive ion mode within the m/z range of 50–800 at the resolution of 140,000 (*m*/*z* 200). Obtained mass spectra were processed using Thermo Xcalibur software (ver. 4.1.31.9).

### 2.3. The Stability Constants

Stability constants (log K) of complexes of salen with Pd^2+^, Ag^+^, Pt^2+^, and Au^3+^ ions were determined as we described in our previous paper [17]. For this purpose, a methanol solution of salen with the concentration of 5.5 × 10^−5^ mol/L and aqueous solutions of metal ions with the concentration of 1 g/L were prepared. Then, to prepare solutions with different molar ratios of ligand to metal ions, each time, a higher amount of metal ions solution was added according to the fixed amount of ligand solution. Finally, the absorption spectra of the obtained solutions were recorded, and stability constants of created complexes of salen with Pd^2+^, Ag^+^, Pt^2+^, and Au^3+^ ions, respectively, were calculated.

### 2.4. Solvent Extraction

The stock aqueous solutions for Pd^2+^, Ag^+^, Pt^2+^, and Au^3+^ metal ions were made by dilutions of pattern metal ion solutions in water, respectively. The organic solution contained *N,N'*-bis(salicylidene)ethylenediamine (L) dissolved in chloroform. The samples for the extraction processes were prepared so that the molar ratio of metal ions to ligand (M:L) was 1:1, 1:5, and 1:10 in single-component metal ion solutions and 1:1 and 1:4 for polymetallic solutions. Small amounts of ammonia solution were added to the metal ion solutions to avoid the creation of hydroxides during the extraction process. All experiments were carried out in graduated test tubes, and the temperature of the processes was 25 ± 0.2 °C. The volume of both phases (aqueous phase and organic phase) was 1100 µL. The parameters of the particular phases in the extraction processes are described in detail in Table 1.

Next, the prepared samples were shaken for 1 h. The equilibrium was established after approximately 15 min by visual observation. Next, it was checked for any changes in the phase volumes, then the phases were separated, and the pH of the aqueous phase was measured. Finally, the metal ion concentration in the aqueous phases was determined by an inductively coupled plasma mass-spectrometer ICP-MS (NexION 300d PerkinElmer, Inc., Waltham, MA, USA).

The extraction percentage (%E_M_) of metal ions was described by the equation as follows:

(1) $${\%E}_{M}=\frac{D_{M}}{D_{M}+\frac{V_{\mathrm{aq}}}{V_{\mathrm{org}}}}\cdot 100\%$$

where D_M_ is the division ratio determined experimentally; V_aq_ is the volume of the water phase [l]; V_org_ is the volume of the organic phase [l] (V_aq_ = V_org_, so V_aq_/V_org_ = 1).

The division ratio is the ratio of the sum of the concentrations of all the substances in the organic phase (Σ[M]_org_) to the sum of the concentrations of all the substances in the water phase (Σ[M]_aq_).

(2) $$D_{M}=\frac{\Sigma {\left[M\right]}_{\mathrm{org}}}{\Sigma {\left[M\right]}_{\mathrm{aq}}}$$

The results were elaborated using a spreadsheet and standard deviation.

### 2.5. The Preparation of Polymer Membranes

**Figure 2:**
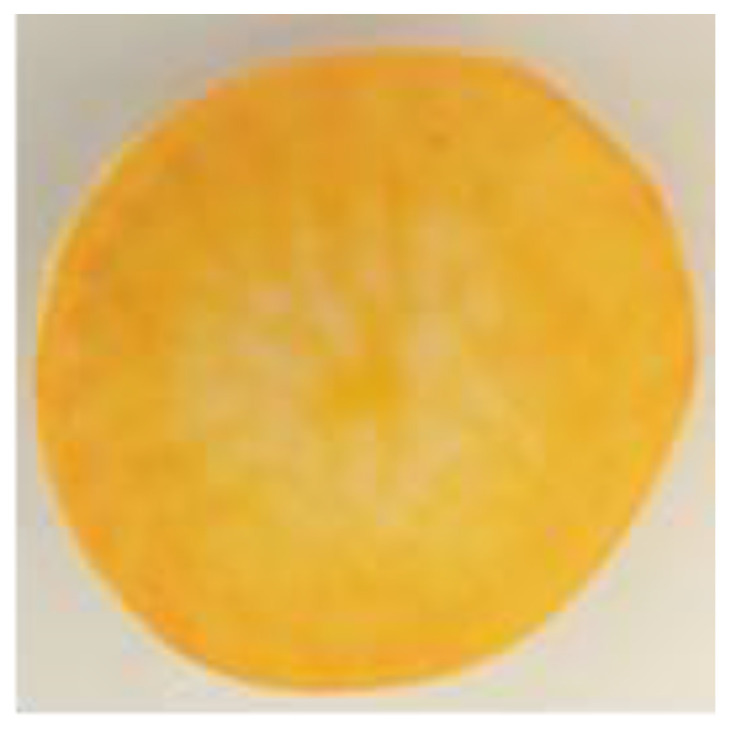
The polymer membrane with 20 wt.% *N,N'*-bis(salicylidene)ethylenediamine before the sorption process.

The polymer membranes (one is shown in Figure 2) were made by pouring on a glass ring the organic solution dissolved in tetrahydrofuran contained a 60 wt.% polyvinylchloride (PVC), 20 wt.% a bis(2-ethylhexyl)adipate (ADO) and also 20 wt.% *N,N'*-bis(salicylidene)ethylenediamine (salen). The solvent was evaporated for 24 h, and the resulting polymer membrane (PM) was conditioned in distilled water for the next 12 h. As a result, the membranes were homogeneous, transparent, flexible, and had good strength. The thickness of the membranes, which were used for precious metal ions such as gold(II), silver(I), palladium(II), and platinum(II) transport, was approx. 0.178 mm.

### 2.6. Sorption and Desorption Experiments

First, the aqueous metal ion solutions were prepared for sorption. In single-component metal ion solutions, the concentration of particular precious metal ions was 80 mg/L, whereas, in polymetallic solutions (MIX), it was 20 mg/L for single metal ions.

The volume of each aqueous solution used as the feed phase was 30 mL. Small amounts of ammonia solution for pH adjustment during the process were added to the metal ion solutions. The circular polymer membranes were immersed for 24 h in beakers with a prepared aqueous solution of metal ions. The contents of the beakers were stirred with a magnetic stirrer. The sorption process was carried out within 24 h. Samples from single-component metal ion solutions and polymetallic solutions (Nos. I–IV and MIX) were taken regularly. The polymer membrane after the sorption process was immersed in 10 mL 5 mol/L HNO_3_ solution for 24 h.

The analysis of the metal ions sorption process onto the membranes with 20 wt.% of *N,N'*-bis(salicylidene)ethylenediamine (L) as a carrier was carried out using Equation (3):

(3) $$q_{t}=\left(\frac{c^{i}-c^{t}}{m}\right)\cdot V$$

where q_t_ is the sorption capacity (mg/g); V is the volume of the solution (l); m is the mass of the sorbent (g); and c^i^ and c^t^ are the analytical metal ion concentrations in the solution at the beginning and after an appropriate time of sorption process (mol/L), respectively.

After 24 h of sorption, the percentage of metal ion removal from the solutions (%R_s_) was also determined (Equation (4)) [23].

(4) $$\%R_{s}=\frac{c^{i}-c^{t}}{c^{i}}\cdot 100\%$$

While the desorption efficiency (%R_des_) was calculated using Equation (5).

(5) $$\%R_{\mathrm{des}}=\frac{c^{t}}{c^{a}}\cdot 100\%$$

where c^a^ refers to the initially sorbed metal concentration during the desorption processes [23,24].

The metal ion concentration in the aqueous phases after sorption and desorption was determined with an inductively coupled plasma mass-spectrometer ICP-MS (NexION 300d PerkinElmer, Inc., Waltham, MA, USA).

## 3. Results

### 3.1. The Stability Constant

**Figure 3:**
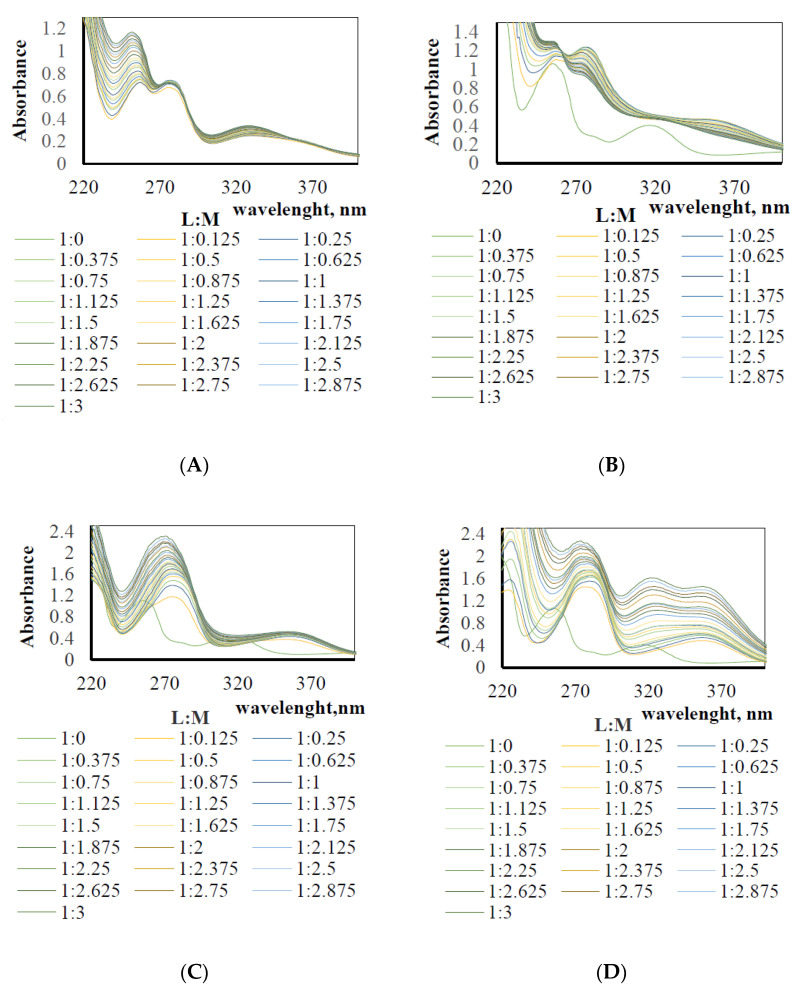
The absorption spectra of the investigated systems (complexes of salen: with (**A**). Pd^2+^, (**B**). Ag^+^, (**C**). Pt^2+^, and (**D**). Au^3+^ ions, respectively.

The recorded absorption spectra of the investigated systems are presented in Figure 3.

Presented spectra show that complexation reactions between salen and the chosen metal ions occur when adding progressively higher amounts of metal ion solutions. Shapes of the spectra of systems with different L:M molar ratios changed in comparison to the spectra of clear ligand or clear metal ions. Maximum absorption of the ligand solution and Pd^2+^, Ag^+^, Pt^2+^, and Au^3+^ ion solutions in the UV region were equal to 255 nm, 275 nm, 272 nm, and 275 nm, respectively. In the spectra of salen with Pd^2+^, Ag^+^, and Pt^2+^, isosbestic points were also clearly visible.

Based on the above absorption spectra, the stability constants of the complexes of the chosen metal ions with salen molecules were calculated (Table 2).

The most stable complexes were formed by salen (L) with palladium (log K_1_ 5.54) and platinum ions (log K_1_ 5.48) involving one ligand molecule and one metal ion (type 1:1). Stability constants of complexes for these metal ions (L:M) decreased in the following order: type 1:1 > type 1:2 > type 1:3. One valent metal ion (Ag^+^) formed with (L) the most stable complexes, which involved one ligand and two metal ions (type 1:2), whereas three valent metal ions such as gold usually formed complex type 1:3.

### 3.2. Solvent Extraction

The solvent extraction of metal ions (Pd^2+^, Ag^+^, Pt^2+^, or Au^3+^) from single-component solutions using *N,N'*-bis(salicylidene)ethylenediamine (L) was carried out in molar ratios of metal ion to ligand of 1:1, 1:5, and 1:10, respectively. In contrast, the removal of metal ions from polymetallic solutions (MIX 1 and MIX 2) was carried out in the ratios of 1:4 and 1:16. Table 3 shows the results of the extraction percentages in various M:L molar ratios.

All obtained extraction percentages had very high values, which indicates the affinity of *N,N'*-bis(salicylidene)ethylenediamine to bind metal ions from aqueous solutions. There was no visible molar ratio (M:L) dependence on extraction percentage, which confirms the high effectiveness of the compound used as an extractant, even in small concentrations. Still, on the other hand, its selectivity is low. The amount of removed metal ions from mixed solutions is similar for all investigated precious metals. The utilization of *N,N'*-bis(salicylidene)ethylenediamine (L) in classical solvent extraction enables the removal of more than 94% of precious metal ions from the aqueous solutions.

**Figure 4:**
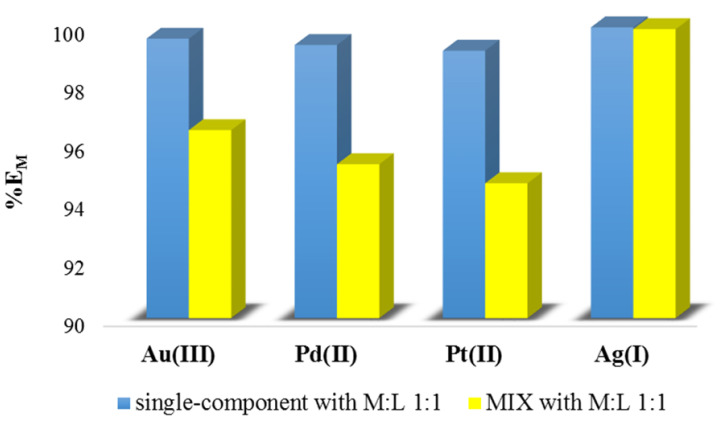
The comparison of metal ion removal from single-component and polymetallic solutions (both with M:L of 1:1) using *N,N'*-bis(salicylidene)ethylenediamine (L) as an extractant. The given values of %E_M_ carry ±0.01.

Figure 4 presents the results of metal ion removal from single-component and polymetallic solutions, both with an identical M:L ratio of 1:1, using *N,N'*-bis(salicylidene)ethylenediamine (L) as an extractant. It was found that the effectiveness of salen as an extractant decreased when noble metal ions (Au(III), Pd(II), and Pt(II)) were removed from the polymetallic samples. However, in the case of the Ag(I) ions, almost the same extraction percentage was observed, regardless of whether these ions were present in single-component or multi-component samples.

Moreover, the division ratio increased with the increase in the percentage extraction. The highest division ratio for the single-component solution was 2074.55 for Ag(I) ions (M:L molar ratio 1:1), and the lowest D_M_ was 13.26 for Pd(II) ions (M:L molar ratio 1:10). In the case of the polymetallic solution (MIX), the lowest D_M_ was for the Pt(II) ions, and the highest for Ag(I) in both M:L ratios.

### 3.3. Sorption and Desorption Experiments

**Figure 5:**
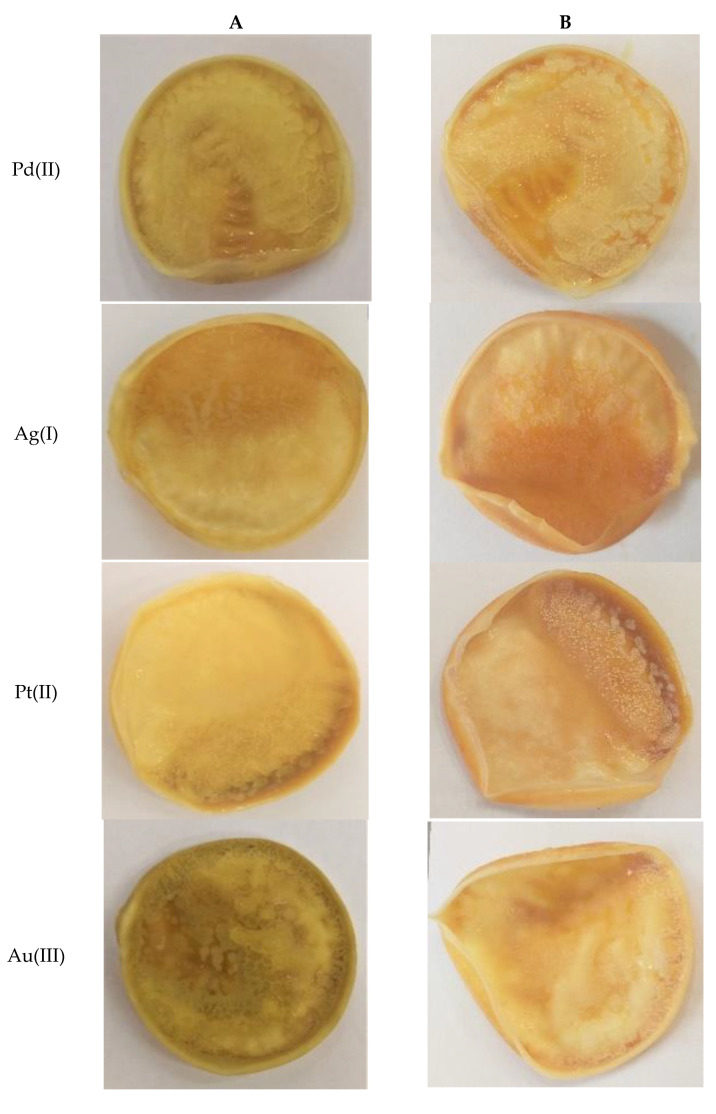

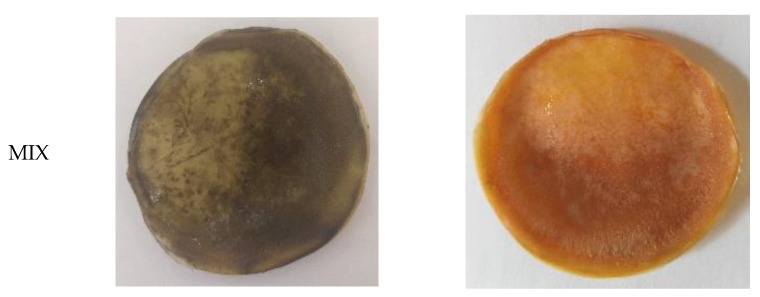
The membranes containing 20 wt.% of *N,N'*-bis(salicylidene)ethylenediamine after sorption (**A**) and desorption (**B**).

The first process for the precious metal ion removal from the aqueous solution was sorption, and then desorption of Pd^2+^, Ag^+^, Pt^2+^, and Au^3+^ ions from the surface of the membranes containing *N,N'*-bis(salicylidene)ethylenediamine (L) as an ion carrier was conducted. The figure below shows the polymer membranes after the sorption (Figure 5A) and desorption (Figure 5B) processes. The photos show massive changes on the surface of the membranes in comparison to a pure membrane (Figure 2). These were caused by the deposition of complexes of precious metal ions created together with molecules of salen contained in the structure of the membranes.

**Figure 6:**
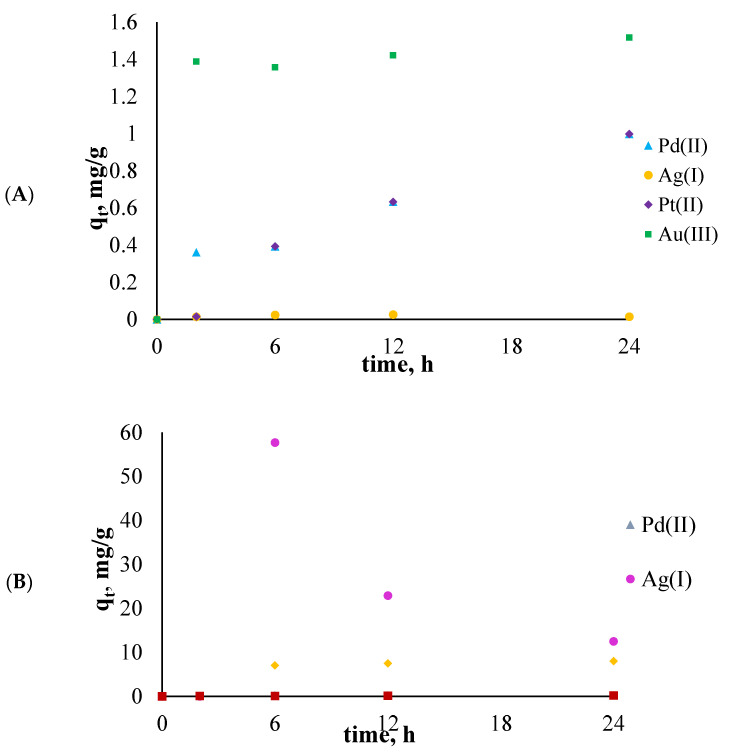
The changes in the sorption capacity of the polymer membranes with 20 wt.% salen as an ion carrier during sorption: (**A**) for single-component metal ions solutions, (**B**) for the polymetallic solution. The given values of q_t_ carry ±0.02.

As a result of the conducted experiments, the sorption capacity (q_t_, mg/g) of the investigated polymer membranes was calculated, as shown in Figure 6. The q_t_ describes the amounts of metal ions adsorbed on the surface of the polymer membrane over a given time.

In Figure 6A,B, some differences can be noticed. First of all, the values of the sorption capacities for the polymetallic solution were larger by two orders of magnitude. For the single component solutions, the sorption capacity over the whole process was the largest for Au(III) and the smallest for Ag(I) ions, respectively, whereas for the polymetallic solution, the exact opposite was observed. The qt obtained for platinum ions was similar for both solutions.

In single-component solutions, the qt decreased in the following order: Au(III) > Pt(II) > Pd(II) > Ag(I), which is the same as the order of electronegativity of the investigated metal ions: Au(III) 2.54 > Pt(II) 2.28 > Pd(II) 2.20 > Ag(I) 1.93. The greater electronegativity of the metal ion caused the greater affinity of this metal for binding to the ligand; thus, the qt of Au(III) was the largest.

On the other hand, in the polymetallic solution, the order of the values of qt was connected with the valence of investigated elements and decreased in the following order: Ag(I) > Pt(II) > Pd(II) > Au(III). Thus, the Ag(I) ions represent the greatest competition for the metal ions present in the solution because they only need one electron to return to the basic state.

**Figure 7:**
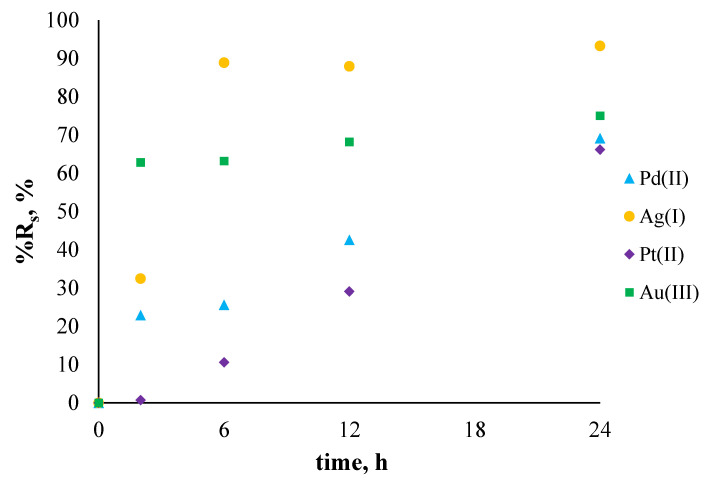
The percentage recovery (%R_s_) of metal ions from single-component solutions using polymer membranes containing salen. The given values of %R_s_ carry ±0.01.

**Figure 8:**
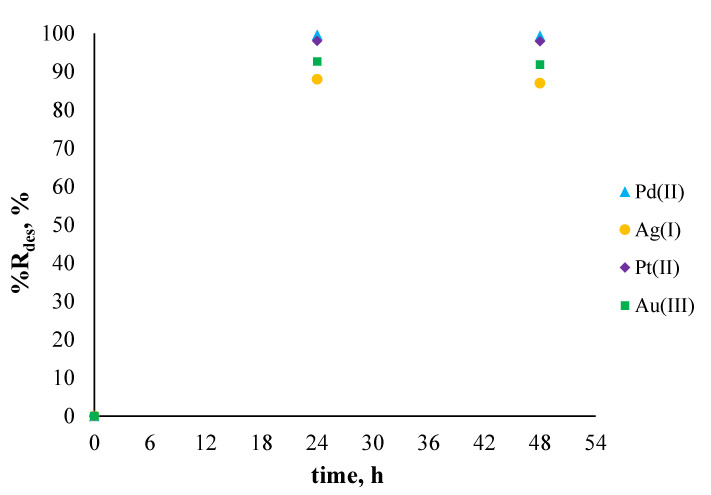
The sum of desorbed metal ions after 24 and 48 h from the surface of membranes previously used to absorb metal ions from single-component solutions. The given values of %R_des_ carry ±0.01.

Figure 7 shows the percentage of removal of noble metal ions from single-component aqueous solutions (%R_s_) during the sorption process conducted after 24 h, while the efficiency of the desorption process (%R_des_) was conducted for 48 h and is shown in Figure 8.

The percentage of noble metal ion removal from the single-component solutions for all metal ions increased with time. In the case of sorption of Au(III) and Ag(I) ions, equilibrium was already reached after 12 h, whereas Pt(II) and Pd(II) ions needed more time. The order of %R_s_ is as follows: Ag(I) > Au(III) > Pd(II) > Pt(II).

After sorption, the opposite process (i.e., desorption) was conducted. For desorption of Pd(II), Ag(I), Pt(II), and Au(III) ions from the surface of polymer membranes containing the investigated ligand (salen), the solution of 5 mol/l of nitric acid was used. Figure 8 presents the percentage of metal ion desorption as the sum of previously adsorbed metal ions on the surface of polymer membranes.

Figure 8 shows that all previously sorbed metal ions on the surface of polymer membranes containing *N,N'*-bis(salicylidene)ethylenediamine were transferred into a solution of nitric acid. It should be noted that the volume of the solution used for desorption was three times smaller (10 mL) compared to the one used for sorption. After 24 h of desorption, almost 100% of all metal ions adsorbed. This confirms that by using investigated membranes containing salen as an ion carrier, it is possible to successfully adsorb metal ions from an aqueous solution on the surface of the membrane and recovered them to another solution.

**Figure 9:**
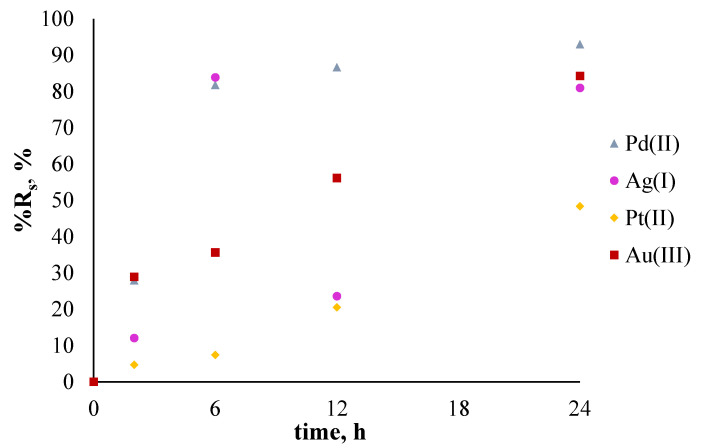
The percentage recovery (%R_s_) of metal ions from the polymetallic solution using a polymer membrane containing salen. The given values of %R_s_ carry ±0.01.

The percentage of noble metal ion removal (%R_s_) from the polymetallic solution for all metal ions increased with time (Figure 9).

The percentages of noble metal ion removal after 24 h of sorption from polymetallic solution (%R_s_) were 92.96%, 84.26%, 80.94%, and 48.36% for Pd(II), Au(III), Ag(I), and Pt(II) ions, respectively.

**Figure 10:**
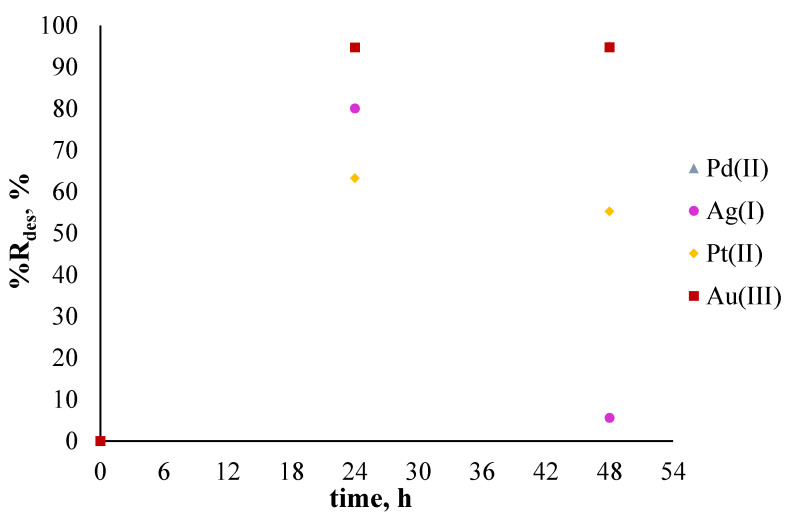
The sum of desorbed metal ions after 24 and 48 h from the surface of membranes previously used to absorb metal ions from a polymetallic solution. The given values of %R_des_ carry ±0.01.

Figure 10 presents the results of desorption.

The highest percentage recovery was observed after 24 h of desorption for Pd(II) (95.03%), then for Au(III) (94.75%), Ag(I) (80.06%), and Pt(II) (63.25%). Compared to desorption conducted after the adsorption of metal ions from single-component solutions, the %R_des_ was lower, and desorption was more complicated, likely because of the presence of the mixtures of salen complexes with investigated metal ions on the surface of the membranes.

As a result of the conducted experiments (Table 4), the highest percentages of noble metal ion recovery from single-component solutions were observed for Ag(I) (93.23%—sorption, 88.04%—desorption) and Au(III) (74.99%—sorption, 92.72%—desorption) ions, whereas for polymetallic solutions, the highest values were observed for Pd(II) (92.96%—sorption, 94.81%—desorption) and Au(III) (84.26%—sorption, 94.72%—desorption).

### 3.4. Mass Spectrometry

**Figure 11:**
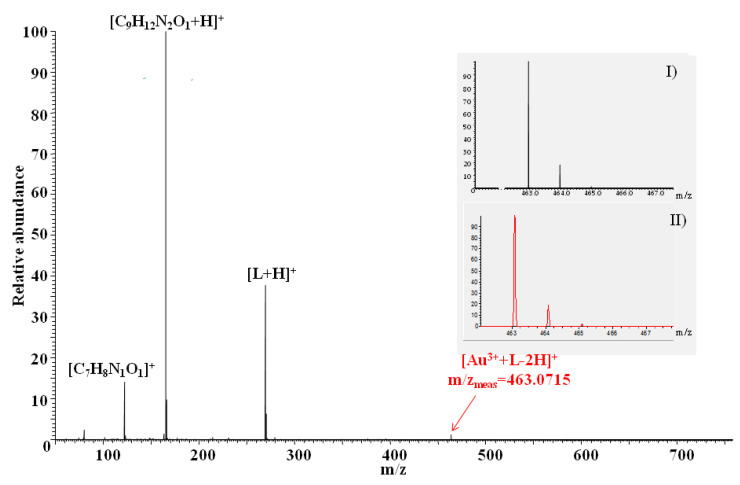
ESI-HRMS spectrum recorded for the sample of the separated organic phase after liquid–liquid extraction containing *N,N'*-bis(salicylidene)ethylenediamine (L) and Au^3+^ ions. Inset shows the isotopic pattern for [Au^3+^+L-2H]^+^ ions; (**I**) experimental, (**II**) theoretical. Unassigned minor signals correspond to ions not relevant to this study (e.g., formed by solvent molecules).

**Figure 12:**
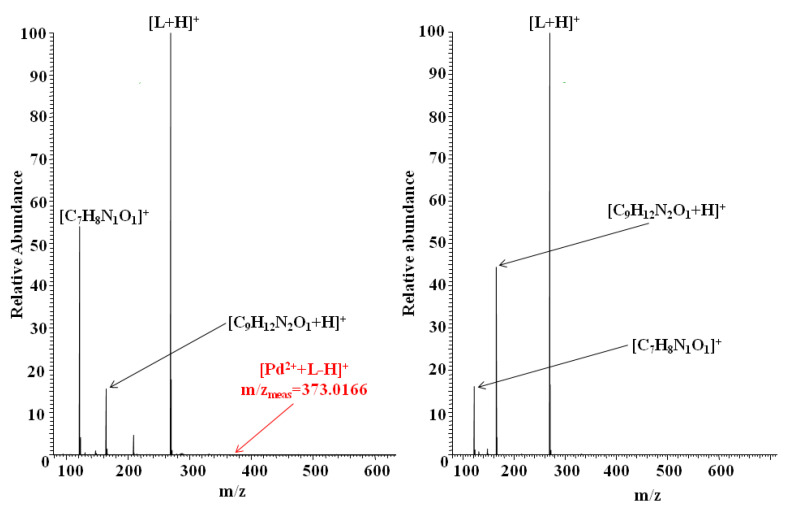
ESI-HRMS spectra recorded for the samples of the separated organic phases after liquid–liquid extraction containing *N,N'*-bis(salicylidene)ethylenediamine (L) and Pd^2+^ (**left**) or Pt^2+^(**right**) ions, respectively. Unassigned minor signals correspond to ions not relevant to this study (e.g., formed by solvent molecules).

High-resolution mass spectrometry (HRMS) characterized by high accuracy of mass measurement (more precisely, the mass-to-charge ratio of ions, *m*/*z*) [25] was used to examine the elemental composition of complexes formed in separated organic phases obtained after liquid–liquid extraction experiments (described in detail in Section 2.4) by *N,N'*-bis(salicylidene)ethylenediamine with noble metal ions such as Au^3+^, Pd^2+^, and Pt^2+^. In all experiments, ionization of molecules was performed by the utilization of the electrospray ionization method (ESI) because ESI, in the case of most small molecules/systems, allows their direct “transfer” from solution to the mass spectrometer without changing their structure [26]. ESI HRMS was previously successfully used to study the elemental composition and charge of complexes formed by salen with copper(II), zinc(II), and nickel(II) ions after the solvent extraction processes [17]. However, it is necessary to emphasize that ESI-HRMS does not allow for the detection of neutral complexes present in the solutions (which cannot be ionized under the experimental conditions) or those that may become neutral as a result of ionization (in ESI, ionization usually occurs as a result of attaching one or more protons to the molecules). ESI-HRMS spectra of the analyzed samples are shown in Figure 11 (samples containing Au^3+^) and Figure 12 (samples containing Pd^2+^ or Pt^2+^), while the ESI-HRMS data of the main ions found are presented in Table 5.

The results of the ESI HRMS experiments performed for all analyzed samples showed the formation of a series of similar, singly charged ions related to the presence of salen such as protonated salen molecules [L + H]^+^, salen fragments ions [C_7_H_8_N_1_O_1_]^+^, and [C_9_H_12_N_2_O_1_ + H]^+^. The generation of identical fragment ions of salen was also observed in the case of ESI HRMS experiments conducted for organic phases resulting from classical liquid–liquid extraction containing *N,N'*-bis(salicylidene)ethylenediamine and copper, zinc or nickel ions [17]. It is most probably related to partial L molecule decomposition (i.e., in the solution, during ESI experiments or compound storage). In the case of samples containing salen and gold(III) ions, an additional, not very intensive signal at m/z = 463.0715 was observed (Figure 11), which can be assigned to [Au^3+^ + L-2H]^+^ ions. Given the high mass accuracy of the HRMS mass spectrometry, there can be no question as to the elemental composition or charge of the ions generated. The process of formation of such mononuclear complexes must be associated with the detachment of two protons from the ligand L molecules (most likely from hydroxyl oxygen atoms). In the case of samples containing palladium ions, the formation of similar mononuclear complexes with the formula [Pd^2+^ + L-H]^+^ was also observed (Figure 12, left spectrum, minor signal at m/z = 373.0166), which must be accompanied by the loss of a single proton from the *N,N'*-bis(salicylidene)ethylenediamine molecules. Interestingly, no corresponding ion formation was noted in the case of samples containing platinum ions (Figure 12, right spectrum). Based on the current and previous results (experiments performed for copper, zinc, and nickel ions, where the formation of other types of salen/metal ion complexes was also observed, for example, [M^2+^ + 2L − H]^+^, [2M^2+^ + 2L − 3H]^+^, where M = Cu^2+^, Zn^2+^, Ni^2+^) [17], it can be concluded that the properties of noble metal ions significantly affected the elemental composition of the formed species. Certainly, not only is the valence of metal ions important, as different metal ion/salen complexes were identified in the ESI HRMS experiments performed for solutions containing other divalent metal ions: copper, nickel, zinc, and palladium. It is necessary to consider the possibility of generating neutral complexes containing several noble metal ions and/or several salen ligands in the examined solutions, which the applied technique could not detect. Moreover, it is very likely considering the results of liquid–liquid extraction (very high process efficiency).

## 4. Summary

The results show that *N,N'*-bis(salicylidene)ethylenediamine can be used as an effective extractant/carrier in recovering noble metal ions from aqueous solutions using separation processes such as solvent or membrane extraction. For scientists involved in research on the application of the extraction and membrane processes for noble metal ion recovery, the main aim is to find an effective extractant/carrier with a high recovery rate, which is not dangerous to the environment. Table 6 compares the efficiency of selected carriers used to recover noble metal ions with the efficiency of *N,N'*-bis(salicylidene)ethylenediamine used in this study.

Based on the results reported by various scientists, it can be concluded that newly synthesized or commercial extractants/ion carriers can be used to recover noble metal ions (Pd, Ag, Pt, and Au). For example, calix[4]arene derivatives can be effective extractants in the recovery of palladium, silver, and platinum ions from aqueous solutions whereas calix[4]pyrrole derivatives can be used as extractants for palladium ions and as carriers for silver ions. *N*-[*N,N*-di(2-ethylhexyl)aminocarbonylmethyl]glycine (D2EHAG) can also be applied in solvent extraction for the recovery of palladium (98%) and gold (69%) ions, and in membrane extraction for the removal of gold ions (96%). Aliquat 336 is an effective extractant for palladium, platinum, and gold ions. Additionally, an amine derivative of β-diketone can be applied to separate palladium and gold ions by solvent extraction. Moreover, ionic liquids are commonly employed in the separation processes to remove noble metal ions from aqueous solution (e.g., Cyphos IL 101 is a good extractant of palladium, silver, platinum, and gold ion solvent extraction). Fajar et al. used a trioctyl(dodecyl) phosphonium chloride (P_88812_Cl) ionic liquid as an ion carrier in polymer inclusion membrane for the removal of Pd(II) ions with an efficiency of 98% [44]. Boudessocque et al. applied ionic liquids bearing tetrahexylammonium and tetraoctylammonium cations and halide, dicyanamide, thiocyanato, and bis(trifluoromethysulfonyl)imide in the solvent extraction process for removal of Au(III) and Pt(II) ions (efficiency above 90%) [45].

Moreover, sorption is also a commonly used method for removing noble metal ions from aqueous solutions. Vojoudi et al. used modified silica-coated magnetic nanoparticles and silica gel as a sorbent to remove gold, palladium, and silver ions [46]. Tahmasebi and Yamini employed polythiophene-coated Fe_3_O_4_ nanoparticles as a selective adsorbent for magnetic solid-phase extraction of silver(I), gold(III), and palladium(II) [47]. Aliquat 336 can be used as an extractant to remove gold, palladium, and platinum ions by the solvent extraction process and can also be applied as sorbent-impregnated for the selective recovery of gold ions (e.g., Aliquat-336-impregnated alginate capsule (AIAC)) [48]. Huang et al. made four adsorption and desorption cycles with a trithiocyanuric-Zr based MOFs adsorbent (ZT-MOFs) for the removal of gold ions (from 94.5% to 87.5%, depending on the cycle) [49].

Despite many available extractants/ion carriers, none of the analyzed compounds were applied to recover all investigated noble metal ions (palladium, silver, platinum, and gold ions) from an aqueous solution. Moreover, *N,N'*-bis(salicylidene)ethylenediamine) can be used as effective active compounds for both processes (solvent and membrane extraction) for the recovery of palladium, silver, platinum, and gold ions.

## 5. Conclusions

The results of the application of *N,N'*-bis(salicylidene)ethylenediamine to recover noble metal ions (Pd^2+^, Ag^+^, Pt^2+^, and Au^3+^) from model aqueous solutions containing single metal ions or their mixture show that this compound is a very efficient extractant in solvent extraction and a carrier in membrane processes. Based on the absorption spectra of complexes of salen with silver, palladium, platinum, and gold ions, it was found that various types of complexes (different L:M ratios) can be formed in the solution. For example, the values of the stability constants were the highest for complexes of the type M:L of 1:1 in the case of palladium and platinum ions (5.54 and 5.48), whereas in the case of silver ions for M:L of 1:2 (4.40) and gold ions for M:L of 1:3 (4.00), respectively. The application of electrospray ionization high-resolution mass spectrometry allowed us to confirm that salen forms mononuclear complexes with gold and palladium ions in aqueous solutions of the type [Au^3+^ + L-2H]^+^ and [Pd^2+^ + L-H]^+^. However, it should be emphasized that the technique used does not allow for identifying other neutral complexes that certainly form during extraction and membrane separation. The results of the extraction experiments show the very high efficiency of salen as an extractant, both in the case of processes conducted in single-component solutions (extraction percentage reached over 99% for all noble metal ions) as well as in polymetallic solutions (the lowest extraction percentage was over 94%). The results of noble metal ion removal obtained with the polymer membranes containing *N,N'*-bis(salicylidene)ethylenediamine as a carrier were slightly worse as the percentage of sorption (%R_s_) of metal ions from single-component solutions was the highest for silver ions (93.23%) and the lowest for platinum ions (66.13%), whereas, for multi-metal solutions, these values were slightly lower (the highest for Pd(II) at 92.96%, and the lowest for Pt(II) at 48.36%). The critical advantage of membrane processes is the possibility of conducting desorption and, consequently, transferring noble metal ions from the surface of membranes to another solvent. The obtained percentage of desorption (%R_des_) was very high, both for single-component solutions (the highest, i.e., 99%, for Pd(II) ions and the lowest, i.e., 88%, for Ag(I) ions) as well as for polymetallic solution (the lowest, i.e., 63.25% for Pt(II)). Another advantage of the membrane process over classic extraction is the need for much smaller amounts of chemical reagents (salen, organic solvents), which is important for economic and environmental reasons.

## Figures and Tables

**Table 1:** The parameters of the particular phases in the extraction process.

Type of Solution	Metal Ions	Sample	M:L	pH	C_M_ Metal Ion in the Aqueous Phase, [mol/L]	C_M_ Ligand in the Organic Phase, [mol/L]
single component solution	Pd^2+^	1	1:1	8.997	0.009	0.009
		2	1:5	9.015	0.002	0.010
		3	1:10	9.111	0.001	0.010
	Ag^+^	4	1:1	10.247	0.009	0.009
		5	1:5	10.354	0.002	0.010
		6	1:10	10.119	0.001	0.010
	Pt^2+^	7	1:1	8.997	0.005	0.005
		8	1:5	9.214	0.002	0.010
		9	1:10	9.059	0.001	0.010
	Au^3+^	10	1:1	9.132	0.005	0.005
		11	1:5	9.325	0.002	0.010
		12	1:10	9.456	0.001	0.010
polymetallic solution	Pd^2+^	MIX	1:1 (for the sum of all precious metal ions) 1:4 (for single metal ion)	9.285	0.00062	0.0025
	Ag^+^					
	Pt^2+^					
	Au^3+^					
	Pd^2+^		1:4 (for the sum of all precious metal ions) 1:16 (for single metal ion)	9.197	0.00062	0.01
	Ag^+^					
	Pt^2+^					
	Au^3+^					

The given values of pH carry ±0.001.

**Table 2:** The stability constants of the complexes of salen (L) with Pd^2+^, Ag^+^, Pt^2+^, and Au^3+^ ions.

L:M Complex Type	Stability Constants	M			
		Pd^2+^	Ag^+^	Pt^2+^	Au^3+^
		log K			
1:1	log K1	5.54	2.30	5.48	0.52
1:2	log K2	4.60	4.40	4.60	4.63
1:3	log K3	4.00	4.30	4.30	4.00

The given values of log K carry ±0.01.

**Table 3:** The results of extraction percentages in various molar ratios M (Pd^2+^, Ag^+^, Pt^2+^, or Au^3+^):L (*N,N'*-bis(salicylidene)ethylenediamine) for single-component and polymetallic solutions.

Type of Solution	Metal Ions	M:L	The Extraction Percentage, %E_M_ [%]
single component solution	Pd^2+^	1:1	99.35
		1:5	97.39
		1:10	92.98
	Ag^+^	1:1	99.95
		1:5	99.86
		1:10	99.83
	Pt^2+^	1:1	99.15
		1:5	97.80
		1:10	94.98
	Au^3+^	1:1	99.56
		1:5	97.77
		1:10	94.89
polymetallic solution (MIX)	Pd^2+^	1:1 (for the sum of all precious metal ions) 1:4 (for single metal ion)	96.43
	Ag^+^		95.27
	Pt^2+^		94.62
	Au^3+^		99.89
	Pd^2+^	1:4 (for the sum of all precious metal ions) 1:16 (for single metal ion)	96.50
	Ag^+^		96.46
	Pt^2+^		94.86
	Au^3+^		99.90

The given values of %E_M_ carry ±0.01.

**Table 4:** The results of the recovery of precious metal ions (Pd^2+^, Ag^+^, Pt^2+^, and Au^3+^) from single-component and polymetallic solutions using polymer membranes containing *N,N'*-bis(salicylidene)ethylenediamine.

Type of Solution	Metal Ions	The Percentage of Sorption, %R_s_ [%]	The Percentage of Desorption, %R_des_ [%]
single component solution	Pd^2+^	69.11	99.68
	Ag^+^	93.23	88.04
	Pt^2+^	66.13	98.07
	Au^3+^	74.99	92.72
polymetallic solution (MIX)	Pd^2+^	92.96	94.81
	Ag^+^	80.94	80.06
	Pt^2+^	48.36	63.25
	Au^3+^	84.26	94.72

The given values of %R_s_ and %R_des_ carry ±0.01.

**Table 5:** ESI HRMS data of the main ions found in the samples of the separated organic phases after liquid–liquid extraction (performed for samples in which the molar ratio of metal ions to ligand (M:L) was 1:5, L-*N,N'*-bis(salicylidene)ethylenediamine) diluted (1:1) in methanol.

Au(NO_3_)_3_ and L (C_16_H_16_N_2_O_2_)			
***m*** **/*z*_meas_**	***m*** **/*z*_calc_**	**Assignment**	**Mass Error [ppm]**
122.0603	122.0606	[C_7_H_8_N_1_O_1_]^+^	2.46
165.1023	165.1028	[C_9_H_12_N_2_O_1_ + H]^+^	3.03
269.1283	269.1290	[L + H]^+^, (C_16_H_17_N_2_O_2_)^+^	2.60
463.0715	463.0720	[Au^3+^ + L-2H]^+^, (AuC_16_H_14_N_2_O_2_)^+^	1.08
**Pd(NO_3_)_2_ and L** **(C_16_H_16_N_2_O_2_)**			
***m*** **/*z*_meas_**	***m*** **/*z*_calc_**	**Assignment**	**Mass Error [ppm]**
122.0604	122.0606	[C_7_H_8_N_1_O_1_]^+^	1.64
165.1025	165.1028	[C_9_H_12_N_2_O_1_ + H]^+^	1.82
269.1287	269.1290	[L + H]^+^, (C_16_H_17_N_2_O_2_)^+^	1.11
373.0166	373.0168	[Pd^2+^ + L-H]^+^, (PdC_16_H_15_N_2_O_2_)^+^	0.53
**Pt(NO_3_)_2_ and L** **(C_16_H_16_N_2_O_2_)**			
***m*** **/*z*_meas_**	***m*** **/*z*_calc_**	**Assignment**	**Mass Error [ppm]**
122.0604	122.0606	[C_7_H_8_N_1_O_1_]^+^	1.64
165.1025	165.1028	[C_9_H_12_N_2_O_1_ + H]^+^	1.82
269.1288	269.1290	[L + H]^+^, (C_16_H_17_N_2_O_2_)^+^	0.74

**Table 6:** Selected extractants/carriers used in the separation processes (SE, PM) for the recovery of noble metal ions.

*N,N*’-bis(salicylidene)ethylenediamine)					
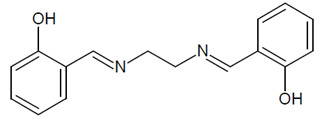					
[%]	Pd(II)	Ag(I)	Pt(II)	Au(III)	Ref.
%E_M_	92.98–99.35%	99.83–99.95%	94.98–99.15%	94.89–99.56%	[This work]
%R_s_	69.11%	93.23%	66.12%	74.99%	[This work]
**2,6-bis(4-methoxybenzoyl)-diaminopyridine**					
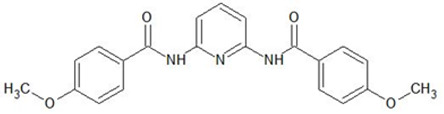					
[%]	Pd(II)	Ag(I)	Pt(II)	Au(III)	Ref.
%E_M_	~99%	~99%	~99%	~99%	[27]
%R_s_	23.82%	94.89%	38.99%	63.46%	[27]
**Ethylenodiamino-bis-acetylacetone**					
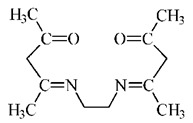					
[%]	Pd(II)	Ag(I)	Pt(II)	Au(III)	Ref.
%E_M_	87–93%	-	-	56–65%	[28]
%R_s_	-	-	-	-	-
**Calix**[4]**arene derivatives**					
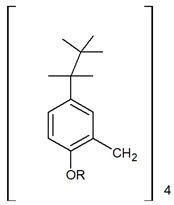					
[%]	Pd(II)	Ag(I)	Pt(II)	Au(III)	Ref.
%E_M_	70.9%	13.3%	64.4%	-	[29]
	99%	-	-	-	[30]
	95%	-	-	-	[31]
%R_s_	-	-	-	-	-
**Calix**[4]**pyrrole derivatives**					
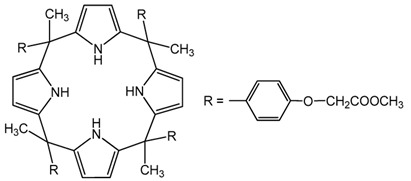					
[%]	Pd(II)	Ag(I)	Pt(II)	Au(III)	Ref.
%E_M_	-	-	-	-	-
%R_s_	-	92.77%	-	-	[32]
	-	80.1%,	-	-	[33]
**D_2_EHAG**					
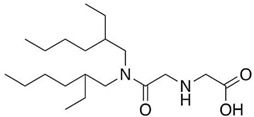					
[%]	Pd(II)	Ag(I)	Pt(II)	Au(III)	Ref.
%E_M_	-	-	-	69%	[34]
	98%	-	-	-	[35]
%R_s_	-	-	-	96%	[34]
**Aliquat 336**					
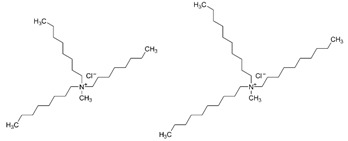					
[%]	Pd(II)	Ag(I)	Pt(II)	Au(III)	Ref.
%E_M_	99%	-	-	-	[36]
	99%	-	-	99%	[37]
	-	-	~100%		[38]
%R_s_	80%	-	-	-	[39]
**Cyphos IL 101**					
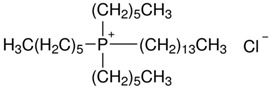					
[%]	Pd(II)	Ag(I)	Pt(II)	Au(III)	Ref.
%E_M_	-	-	~100%	-	[38]
	>90%	93–95%	-	-	[40]
	99.9%	-	-	99.9%	[41]
	-	-	-	98.4%	[42]
	84–90%	-	-	-	[43]
%R_s_	~45%	-	-	-	-

## Data Availability

Not applicable.
